# Identification and Improvement of Hazard Scenarios in Non-Motorized Transportation Using Multiple Deep Learning and Street View Images

**DOI:** 10.3390/ijerph192114054

**Published:** 2022-10-28

**Authors:** Yiwen Wang, Di Liu, Jiameng Luo

**Affiliations:** 1 School of Architecture and Urban Planning, Nanjing University, Nanjing 210093, China; 2 School of Aeronautics and Astronautics, Sun Yat-sen University, Shenzhen 518107, China

**Keywords:** non-motorized transportation, multiple deep learning, hazard scenarios, street view images, seed points spreading + PSPNet algorithm

## Abstract

In the prioritized vehicle traffic environment, motorized transportation has been obtaining more spatial and economic resources, posing potential threats to the travel quality and life safety of non-motorized transportation participants. It is becoming urgent to improve the safety situation of non-motorized transportation participants. Most previous studies have focused on the psychological aspects of pedestrians and cyclists exposed to the actual road environment rather than quantifying the objective safety hazards, which has led to a non-rigorous evaluation of their basic safety situation. An integrated processing approach is proposed to comprehensively and objectively evaluate the overall safety level of non-motorized transportation participants on each road segment. Our main contributions include (1) the universal approach is established to automatically identify hazard scenarios related to non-motorized transportation and their direct causing factors from street view images based on multiple deep learning models; (2) a seed points spreading algorithm is designed to convert semantic images into target detection results with detail contour, which breaks the functional limitation of these two types of methods to a certain extent; (3) The safety situation of non-motorized transportation on various road sections in Gulou District, Nanjing, China has been evaluated and based on this, a series of suggestions have been put forward to guide the better adaptation among multiple transportation participants.

## 1. Introduction

Non-motorized transportation generally refers to the transportation with a travelling speed not greater than 15 km/h, including walking, cycling or using other non-motorized vehicles [1]. Compared with traditional motorized transportation, non-motorized transportation is capable of guaranteeing accessibility, promoting social and cultural inclusion [2] and benefiting physical health [3]. As a travelling mode that does not rely on fossil and electric energy, it also contributes to environmental and ecological protection [4]. However, the road space for non-motorized transportation participants has been continuously squeezed because motorized transportation obtained more spatial and economic resources under the influence of traditional “car-oriented” urban planning strategy. The travelling quality and life safety of non-motorized transportation participants are increasingly being adversely affected. Despite the death toll from road traffic accidents with motor vehicles having declined from 2007 to 2015 in China, deaths from non-motor vehicle accidents have risen rapidly since 2012 [5]. Therefore, there is an urgent need to improve the current non-motorized transportation situation.

The influencing factors of non-motorized transportation participants’ safety have widely discussed by using the type of motor vehicle [6], the duration of signal lights [7], the distraction of the drivers [6], the flags of pedestrian crossing [8], the conformity and group identity of pedestrians [9] as well as their safety awareness and knowledge of traffic [10]. In addition, an increasing number of studies have been evaluating the safety of the road environment from the aspect of overall road environment characteristics [11,12,13,14]. For example: Galanis and Eliou [13] have found that while street furniture such as trees, litter baskets and benches reflect the walkability characteristics of the street to a certain extent, their presence also acts as a barrier to walking on the sidewalk, letting pedestrian to walk on the motorway and reducing the level of road safety. However, these studies mainly rely on in-field manual survey method to investigate the impact factors and assess the degree of traffic hazard [8], which not only takes huge time and economic costs, but is also vulnerable to bad weather and the local social environment [15]. Moreover, these methods may also bring unpredictable danger to the investigators [16]. Thereby, an efficient, convenient and low-cost method for observing the traffic hazard is essential to objectively evaluate the regional safety situation of non-motorized transportation participants.

Big data resources such as social media data, mobile phone signaling, and road surveillance video have recorded a variety of traffic information, providing various perspectives to reflect the safety situation of regional non-motorized transportation participants. Among these data sources, the spatial accuracy of mobile phone signaling data depends on the density of the base station distribution, making it challenging to obtain the acceptable paths of users [17]. Road surveillance video often only reflects information about the people on a particular road section, not the whole area. Compared with the above data sources, street view images have massive potential in studying the regional safety situation of non-motorized transportation participants, with the advantages of broad coverage [18], rich information [19], and the fine-grained observations of both physical environment and social sensing [20]. As a result, street view images have been used to explore the impacts of urban environment characteristics on walking and cycling [21,22,23,24].

Moreover, to study transportation problems effectively and safely, many scholars recently have used machine learning models for target detection, semantic segmentation, image classification and other computer vision operations to extract the required information from a massive amount of street view images [25,26,27,28]. The presence and location information of ground objects of interest in the street view images can be obtained and detected by applying target detection algorithms, such as RetinaNet, Decorrelated Channel Features (LDCF) algorithm, Locally Decorrelated Channel Features (LDCF) algorithm. In contrast with target detection algorithms that can only identify the approximate location of target objects, semantic segmentation algorithms can finely classify each pixel of a street view image. (Yang et al. [27] have used the Pyramid Scene Parsing Network (PSPNet) method to implement semantic segmentation and assess the eye-level of street greenness in Google Street View (GSV) images by calculating the ratio of street vegetation to the whole picture. In addition, some scholars have further performed image classification on the extracted target objects to obtain more refined information [25]. In summary, previous studies have demonstrated the great potential of using street view images and machine learning models in assessing the quality of walking and cycling environments and their impact on pedestrians’ or cyclists’ willingness to travel.

However, conventional road safety quality assessments only focus on static environmental characteristics and often lack the consideration of objective hazards for non-motorized transportation participants. To realistically evaluate their safety situation, it is necessary to observe and count the traffic hazards on each road segment, such as pedestrian-vehicle mixing and pedestrians occupying the motorway, as well as the direct causes of these hazardous behaviors, such as narrow sidewalks and discontinuous sidewalks [29,30]. This study aims to tackle this problem by identifying the most intuitive and objective hazards scenarios in the street view images, with a case study taken in Gulou District, Nanjing City, China. Our objectives are three-fold: (1) proposing an innovative and integrated method for automatically identifying the hazard scenarios (HS) and reason scenarios (RS) of non-motorized transportation based on multiple deep learning models and street view images; (2) analyzing the spatial distribution characteristics of local hazard situation and their causal factors; (3) providing helpful guidance for updating the non-motorized traffic system with a cost-effective investment of land and economic resources.

## 2. Materials and Methods

### 2.1. Study Area

**Figure 1:**
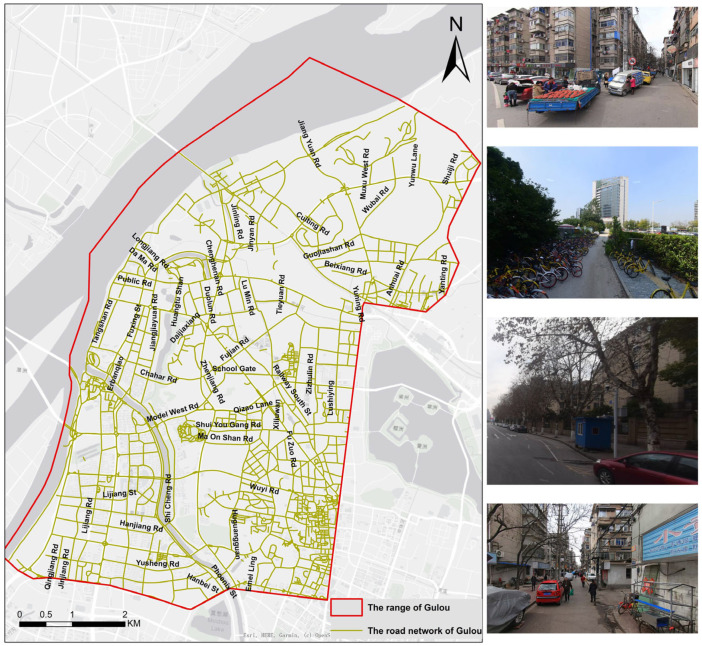
The road network of Gulou District, Nanjing and the common scenarios cropped from street view images.

Gulou District is the core area of Nanjing, Jiangsu Province, China, with a long history and dense population [31], and its road network is shown in Figure 1. The problems of non-motorized traffic safety in the Gulou District are highly representative of many regions in China. First, the area has a complex road network, and roads’ directions appear chaotic, and many branches have no specific space for pedestrians. Secondly, there are many local electric vehicles and motorcycles for commuting and urban logistics services (i.e., express and food delivery), which negatively affect the non-motorized transportation participant [32]. In addition, plenty of parked vehicles, newspaper kiosks, and other obstructions occupy or even completely cut off the sidewalks [33], which causes inconvenience to pedestrians. Moreover, the ageing infrastructure and narrow downtown roads make the safety hazards of non-motorized transportation increasingly prominent. Therefore, Nanjing, Gulou District, demonstrates the renewal and improvement of the non-motorized transportation environment within the old town. 

### 2.2. Hazard Scenarios about Non-Motorized Transportation

**Figure 2:**
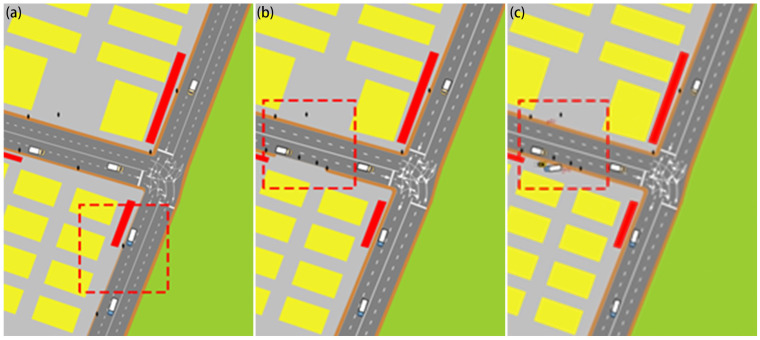
Sidewalks being excessively narrow (**a**), being spatially discontinuous (**b**) and the obstructions occupying the sidewalk (**c**).

Two types of HS are extracted to evaluate the safety situation of non-motorized transportation participants: (1) Hazard Scenario 1 (HS1): pedestrians walking on the motor vehicle lanes in a road environment where there is a clear distinction between the motor vehicle lanes and the sidewalks; (2) Hazard Scenario 2 (HS2): mixing of motor vehicles, non-motor vehicles and pedestrians in the roadway environment where there is no clear distinction between motor vehicle lanes and sidewalks. Moreover, three Reason Scenario (RS) causing HS1 are selected from the perspective of non-motorized transportation participants: sidewalks being excessively narrow, sidewalks being spatially discontinuous, and obstructions occupying the sidewalks as shown in Figure 2. The description of the various categories of study scenarios is shown in Table 1.

### 2.3. Study Data

The street view images used in this study include three types, namely Baidu Map Street view images, Cityscape dataset (https://www.cityscapes-dataset.com, accessed on 12 May 2019), and Apollo dataset (http://apolloscape.auto, accessed on 21 March 2018). The newest updated Baidu Map Street view images are applied to obtain non-motorized transportation information in the study area. In addition, the Cityscape and Apollo datasets are employed to train the semantic segmentation and image classification models, respectively.

#### 2.3.1. The Cityscape Dataset

This dataset contains 5000 street view images mainly collected from 50 different European cities, with the same number of high-quality annotated images containing 19 categories of ground objects in the urban environment. The pixel values and their corresponding types are shown in Table 2. The training, validation, and testing groups comprise 2975, 500, and 1525 images.

#### 2.3.2. The Apollo Dataset

To identify HS1, HS2 or HS1.RS3, the Squeeze-and-Excitation Network (SENet) [34] model, is applied to imitate the human’s judgment mechanism in the image classification tasks. The samples for training SENet are randomly selected from the Apollo dataset. The images in the Apollo dataset are mainly collected in Shanghai, China, which is adjacent to Nanjing, and the two cities have relatively similar social environments. However, this dataset cannot be used directly to train the SENet model but needs to be manually processed into training samples based on a set of explicit criteria, as shown in Table 1. When creating the training samples for HS, take screenshots of the parts of the images that meet the above scenario criteria in Table 1 and save them as positive samples. In addition, screenshots that do not meet these criteria also need to be obtained as negative samples. Finally, three datasets with different amounts have been obtained. There are 1258 positive samples and 1733 negative samples for HS1, 1456 positive samples and 1983 negative samples for HS2, 2317 samples positive and 1055 negative samples for HS1.RS3. For each dataset, 80.00% is for training, 10.00% for evaluation 10.00% for testing.

#### 2.3.3. The Baidu Map Street View Dataset

**Figure 3:**
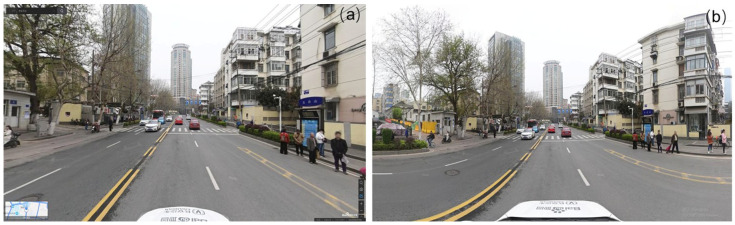
(**a**,**b**) A sample of collected street view image.

Due to strategic and business policies, GSV images are hard to be obtained in China [35]. Baidu Map and Tencent Map are the two major Chinese street-view providers. Baidu Map has an advantage in updated frequency and has covered 95% of Chinese cities and more than 3 million kilometers [35]. Figure 3a shows that the image captured on the Street View map platform only reflects potential conflicts from a specific perspective. As a comparison, the panoramic photo downloaded using the Baidu Maps API, despite a little distortion, provides a complete reflection of pedestrian behavior from front, left, right perspectives, as shown in Figure 3b.

### 2.4. Methods

**Figure 4:**
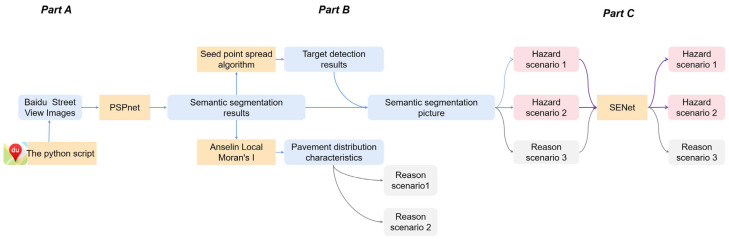
The framework of this study.

There are three parts to the universal approach for automatic identification of HS and RS, and the overall framework is shown in Figure 4.

- Acquisition of street view images and extraction of semantic information. This part mainly describes the street view data acquisition method and introduces the PSPNet, which is applied to perform semantic segmentation.
- Target detection and primary screening for scenarios. This part mainly describes the algorithm to detect target objects based on the seed points spreading algorithm [36] and introduces the way for preliminary screening of HS and RS.
- Extraction of specific scenarios using SENet. This part describes the SENet, which is applied to discriminate real ones from the preliminary screening results.

#### 2.4.1. Street View Image Acquisition and Semantic Information Extraction

(1) Street View image acquisition and semantic information extraction

**Figure 5:**
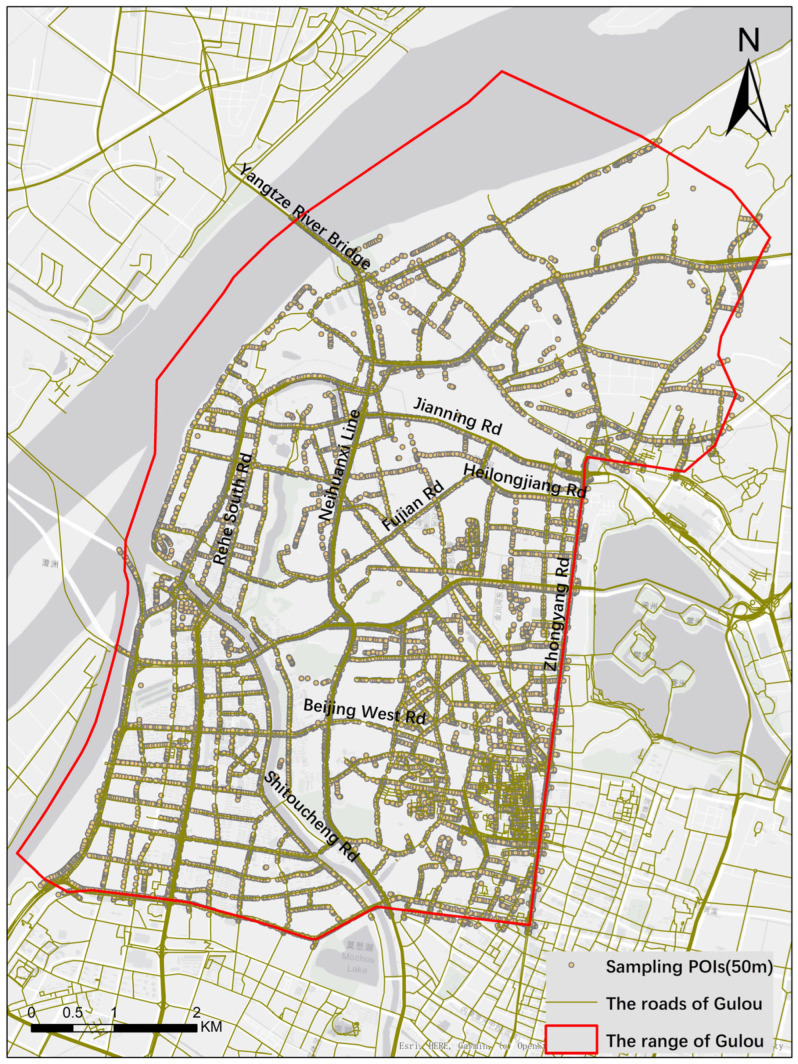
The sample points for downloading street view images of Gulou.

The fishnet map with the WGS84 coordinate system is firstly obtained for Gulou District, and the sampling distance is set to 5.0 m. Next, delete the fishnet label points whose distance to the roads is greater than 5.0 m and the remaining fishnet label points are shown in Figure 5. Check whether points of interest (POIs) represent the street view images around the remaining fishnet label point and record its ID and coordinates. According to the statistics, there are 13,534 street view images on all roads in Gulou District. In this paper, to compare the information difference between street view images obtained by the two routes, we used these two methods to acquire all 13,534 street view images twice.

(2) Semantic segmentation for street view images based on PSPNet

**Figure 6:**
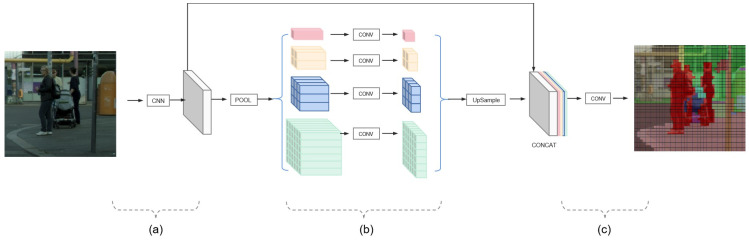
(**a**–**c**) The structure of the PSPNet model.

This study applies the Pyramid Scene Parsing Network (PSPNet) [37], an improved semantic segmentation network based on the Fully Convolutional Network [38], to identify the category of each pixel. This model integrates the local spectral information of pixels and the global overall structural information to extract abstract features. Furthermore, in the delicate segmentation problem, a breakthrough has also been made to identify cars better and eliminate their effects on pixel layers. The model architecture is shown in Figure 6. 

The structure of the PSPNet model used in this paper can be divided into three modules: (a) residual neural network module, which performs feature extraction on the input image using convolution (Conv) layer and residual structure; (b) pyramid pooling module, which performs global averaging pooling operations on the input feature maps at different scales to obtain deep and shallow features of the image, and then adjusts the number of feature maps’ channels to one-fourth of the original’s with following Conv layer; (c) outputting module, which up-samples and combines the output of the pyramid pooling module and feeds the new feature map into the next convolution layer to obtain a single channel image with the same shape of the input image [37]. The specific parameter setting of convolution layers and pooling layers in each part are shown in Table 3. The activation function applied in these convolution layers whose kernel size is larger than 1 × 1 is Rectified Linear Unit (ReLU).

(3) Identification method of HS1.RS1 and HS1.RS2

**Figure 7:**
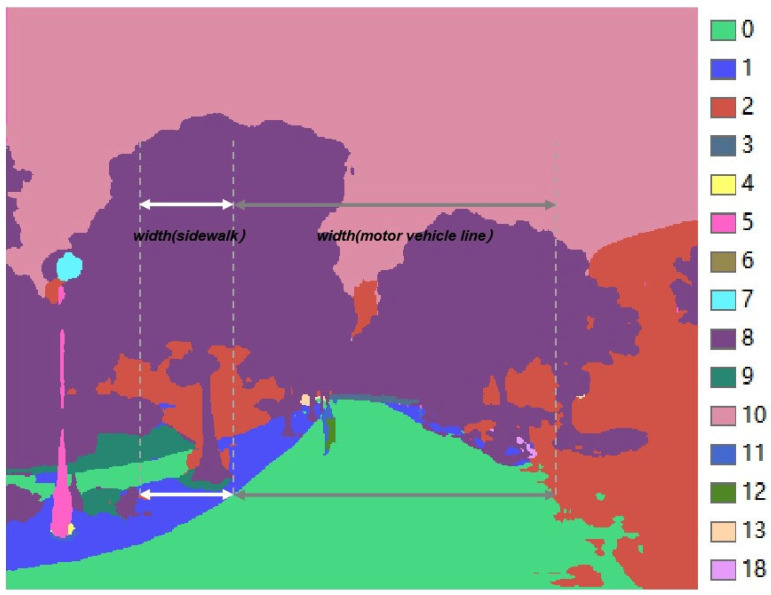
The calculation method of the ratio of sidewalk’s width relative to road’s width.

Based on semantic segmentation results output by PSPNet, two categories of RS can be identified: sidewalks being excessively narrow and spatially discontinuous. Before determining these RS, we first need to judge the spacious degree of the sidewalks based on the semantic segmentation images [38]. Li et al. [39] have evaluated this index with the ratio of the sidewalk to the road. However, this method does not always reflect the situation since sidewalks are often covered by motor vehicles, bicycles, pedestrians and other ground objects. In contrast, the relative width of the sidewalk can be more effectively evaluated using the ratio of the sidewalk’s width close to the road’s width in the horizontal direction, as shown in Figure 7.

To identify HS1.RS1, classify the relative widths of all semantic segmentation images into 10 grades to analyze the spatial distribution characteristic of sidewalks’ resources in the study area.

(1) $$w_{i}^{k}=w_{si}^{k}/w_{mi}^{k}$$

where $w_{i}^{k}$ represents the width ratio of the sidewalk relative to the motorway in the ***i*th** row of road section k’s segmentation result;$\;w_{si}^{k}$ represents the number of pixels belong to sidewalk in the ***i*th** row; $w_{mi}^{k}$ represents the number of pixels belong to motorway in the ***i*th** row.

(2) $$w^{k}=INT\left(\underset{i=1,2,\ldots ,n}{\max}\left(w_{i}^{k}\times C\right)\right)$$

where $w^{k}$ represents the grade of sidewalk in road section k, ***INT*** is integer operation, and ***C*** represents Constant 10. When the grade of a sidewalk is less or equal to 1, the sidewalk of this road is generally invisible in the forward, left, and right view, which is not part of HS1.RS1. In addition, when the grade is greater than 3, the sidewalk is generally spacious. Therefore, sidewalks with a grade equal to 2 or 3 can be regarded as HS1.RS2.

In terms of HS1.RS2, the sidewalk is spatially discontinuous, as evidenced by the fact that sidewalks are spacious in some sections but relatively narrow or even interrupted directly in the surrounding road sections, so pedestrians could not have a continuous and comfortable walking experience on the sidewalk. Relevant studies calculated the standard deviation of the proportion of sidewalks in multiple street view images as an evaluation index [17]. In this study, the Anselin Local Moran’s I is firstly calculated to obtain the LISA (Local Indications of Spatial Association) [40] figure to present the spatial distribution of sidewalks’ relative widths in the study area. The Anselin Local Moran’s I analysis, also known as clustering and outlier analysis, is a bottom-up systematic clustering in theory. The Anselin Local Moran’s I algorithm calculates the Moran’s I index and z-score for each input point.

(3) $$S^{2}=\frac{1}{n}\overset{n}{\underset{k}{\sum }}{\left(w^{k}-\bar{w}\right)}^{2}$$

(4) $$Z^{k}=w^{k}-\bar{w}$$

(5) $$Z^{j}=w^{j}-\bar{w}$$

(6) $$I^{k}=\frac{Z^{k}}{S^{2}}\overset{n}{\underset{j\neq k}{\sum }}Z^{j}*weight^{kj}$$

where $S^{2}$ represents the deviation of the relative widths of sidewalks on road section i’s neighboring roads; $Z_{i}$ is the deviations of sidewalk’s relative width in this road section from the mean values of neighboring roads; $Z_{j}$ refers to the deviation of the relative width of the sidewalk of a particular road section, which is neighboring to section i; $I_{i}$ represents the local Moran’s I of the road section i. Furthermore, the ${weight}_{ij}$ represents the spatial weight value. The cluster/outlier patterns based on $I^{k}$ could be classified as HH cluster, LL cluster, LH outlier and HL outlier. Among them, the LH outlier, where the variables of the central spatial unit have low values and the variables of the neighboring units have high values, can be regard as HS1.RS2.

#### 2.4.2. Target Detection and Primary Screening for Hazard Scenarios 1, 2 and Reason Scenarios 3

Since a complete street view image may present multiple hazard scenarios, detection for target objects and cropping potential scenarios are required here for subsequent processing and judgment.

(1) Target detection based on seed points spreading algorithm

**Figure 8:**
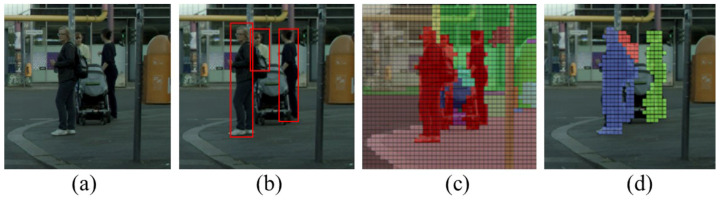
The comparison of the original image (**a**), target detection image output by YOLO (**b**), the semantic segmentation image output by PSPNet (**c**) and target detection image output by seed points spreading algorithm (**d**).

Standard target detection algorithms such as the series of You Only Look Once (YOLO) [41,42] and Single Shot Multi-Box Detector (SSD) models [43], can quickly locate objects of the target from an ordinary image, as shown in Figure 8a. The results output by these models is presented as the bounding boxes of the detected targets, as shown Figure 8b. In contrast, the target detection method applied in this study can directly locate non-motorized transportation participants based on a semantic segmentation result, shown Figure 8c. Furthermore, the output of the target detection algorithm can be a pixel-by-pixel classification result, as shown Figure 8d, or it can also be translated into the bounding boxes, such as Figure 8b. The core idea of the algorithm is as follows:

(1) Create three collections. One is for storing target objects, namely “object list”. Another is for storing seed points, namely “seed list”. In addition, the last is for keeping the points to skip, namely the “skip list”. Next, the semantic segmentation image is sampled at equal distances with a sampling distance of 10 pixels, and the sampled pixel points are put into a “seed list”.
(2) The following steps do not involve equidistant sampling operations. Check the eight neighborhood points with coordinates of (i ± 1, j), (i, j ± 1), (i ± 1, j ± 1) of every seed with coordinate of (i, j) in “seed list”. If the neighborhood point is in the “skip list”, skip it. If it is not in the “skip list”, judge whether it belongs to the target class. If so, add it to the “seed list”. Finally, put every neighborhood and origin seed points into the “skip list”.
(3) Fetch the seed point in a no-return manner from the “seed list”. If the seed point’s pixel (i, j) belongs to the target class and has not been a part of the existing object, create a new entity in the “object list” and regard the pixel (i, j) as the first point of this new object.
(4) Repeat steps 3 and step 2 until no seed point in the “seed list”. Give a new unique value for each object in the “object list”, and the result is shown in Figure 8d.

(2) Screening and cropping suspected hazard or reason scenarios

Based on the target detection results obtained in the previous stage, the specific scenarios would be initially screened according to the location of the non-motorized transportation participants, including pedestrians and cyclists. The screening criterion is set for each type of scenario, as shown Table 4.

The screening criterion in Table 4 involves judging the position relationship “intersection”. This study sets this relationship to a 10% or more intersection of union (IOU) ratio between the two outer rectangles of target objects. In addition, the lowermost profile refers to the part of the target object within the lowermost tenth of the vertical direction. In addition, the outermost profile refers to the amount of the target object within the uppermost or lower tenth of the vertical direction and the leftmost or rightmost part of the horizontal direction. After screening the combinations of target objects, we would calculate the coordinate ranges of these objects to obtain a typical external rectangle, which is then cropped and saved into the corresponding dataset.

#### 2.4.3. Extraction of Hazard Scenarios 1, 2 and Reason Scenarios 3 Using SENet

(1) Binary classification based on the SENet model

**Figure 9:**
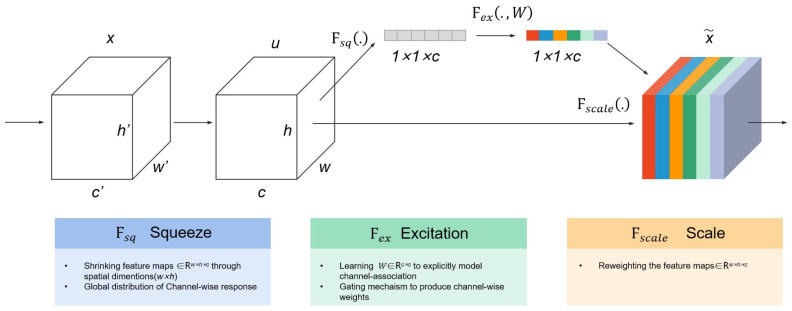
The illustration of squeeze and excitation (SE) blocks.

Recently, multiple deep learning models have been applied to classification tasks because of their outstanding feature extraction ability, such as AlexNet [43], VGG [44], Google Net [45] and ResNet [46,47]. SENet model won the ImageNet 2017 competition for the classification task [34]. The main advantage of the SENet model is to mine the relationship among features in various channels to automatically learn the importance of different information channels using squeeze and excitation (SE) blocks, as shown Figure 9. Therefore, SE block can be combined with ResNet, Inception network and other deep learning models to improve the models’ performance.

SE block performs feature recalibration on the feature maps output from any transformation. First, squeeze the features map U by aggregating these feature maps across spatial dimensions H × W to produce a channel descriptor with the shape of 1 × 1 × c, where c equals the number of channels. Next, an excitation operation is performed to learn the importance of each track, in which sample-specific activation, learned for each channel by a self-gating mechanism based on channels’ dependence, governs the excitation of each channel. Finally, the feature maps U are re-weighted to generate the output result, then fed directly into subsequent layers. Moreover, the SE block does not need to be parameterized because the size of the pooling window used in the squeezing operation equals the size of the input feature maps. The SENet also chooses Resnet as the backbone network for feature extraction, and the network structure and model parameters are different from that of PSPnet, which is configured as in Table 5. The activation function applied in these convolution layers whose kernel size is larger than 1 × 1 is ReLU.

(2) Image processing for crosswalk detection

To avoid misclassifying the expected behavior, walking on the zebra lines, such as HS1, a simple method is applied to make the zebra lines more evident in the image. Next, the extracted zebra lines would be used as part of the input information of the SENet model to reduce the difficulty of removing features.

**Figure 10:**
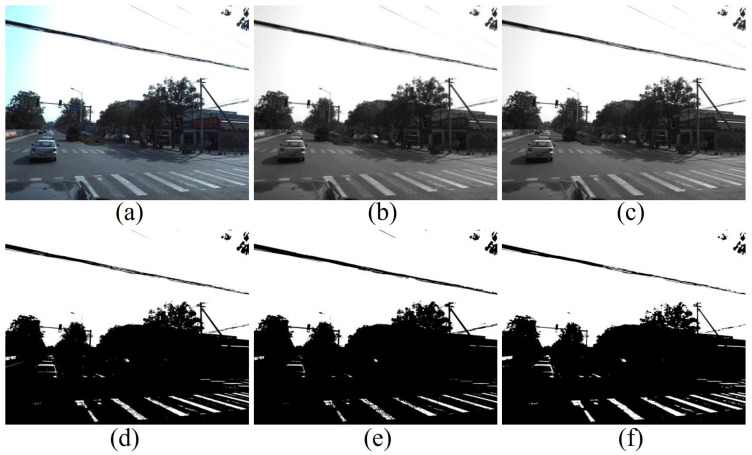
The comparison of origin image (**a**), grayscale image (**b**), grayscale image after Gaussian filtering (**c**), binarization image (**d**), binarization image after erosion (**e**) and binarization image after dilation (**f**).

The first step is to convert the RGB-channel image, as shown Figure 10a, to a single-channel grayscale image based on the OpenCV module. The conversion result is shown Figure 10b. Next, the converted result is processed with Gaussian filtering, which means that each pixel’s value in the image is taken as the weighted average of its neighboring pixels’ values. After the Gaussian filtering process, the portion of zebra lines is still not prominent in the whole image, as shown Figure 8c, mainly because there are too many extra elements. To make the characteristics of the image more streamlined, the grayscale image is processed with the following method:

(7) $$g\left(x\;,y\right)=\{\begin{array}{c} 1,g\left(x,y\right)\geq T;\\ 0,g\left(x,y\right)<T. \end{array}$$

where ***T*** is the threshold value of binarization, after a series of attempts, it is found that a constant threshold value cannot effectively binarize massive street view images. Therefore, we calculate threshold ***T*** for each image by traversal way to make the proportion of pixels whose value equal to 0 in the binarization result exceeds 80%. The result is shown in Figure 10d. After the binarization process, the morphological operations are performed. In this case, i.e., erosion and dilation, and the results are shown in Figure 10e,f. Finally, the zebra lines are still not completely separated from other objects. Still, they are relatively evident in the whole grayscale image, which facilitates the SENet model to understand the scenarios information in images.

## 3. Results and Discussions

### 3.1. PSPNet Semantic Segmentation Experiment

**Figure 11:**
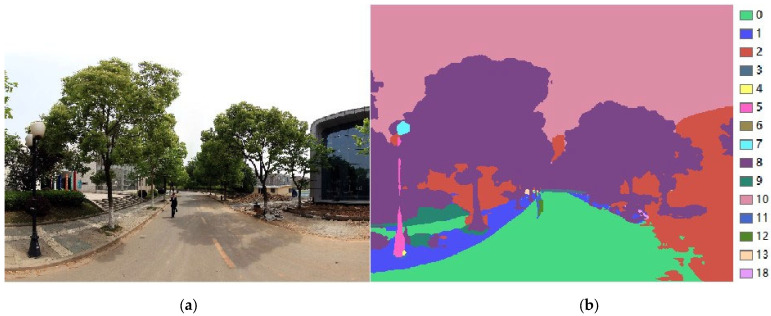
A sample of origin street view image (**a**) and the semantic segmentation result (**b**).

An NVIDIA GeForce GTX 1660 Ti Graphics Processing Unit (GPU) has been used to speed up the training process. During the training process of the PSPNet model, the batch size is set to 2, and 60,000 iterations for training have been performed. The Momentum Optimizer is used as the optimizer, while the learning rate is set to 0.001 and the momentum rate is set to 0.9. As a result, the PSPNet model achieves an average IOU of 0.76 on the Cityscape validation dataset, which is a promising semantic segmentation result. Using the wholly trained PSPNet model, all street view images collected in the study area have been segmented. The comparison of the origin street view image and its semantic segmentation result is shown in Figure 11.

Using PSPnet to segment 27,068 street view images semantically, it is found that there are significant differences between the street view images obtained by the two routes in reflecting the characteristics of the road environment. Of the 13,534 street view screenshots obtained in the forward direction, 9686 photos cannot identify the sidewalks. In contrast, in the panoramic images obtained by the second route, only 1400 images cannot identify sidewalks. This discrepancy is because the sidewalks in the Gulou District are visually similar to motorways, so PSPnet incorrectly identifies the sidewalks as buildings or motorways when processing the screenshots in the forward direction. In contrast, the sidewalk objects on the left and right sides are adequately recorded in the panoramic view, so PSPnet tends to identify the sidewalk’s presence effectively. In summary, we choose 13,534 panoramic images as the basis for identifying sidewalks’ levels.

**Figure 12:**
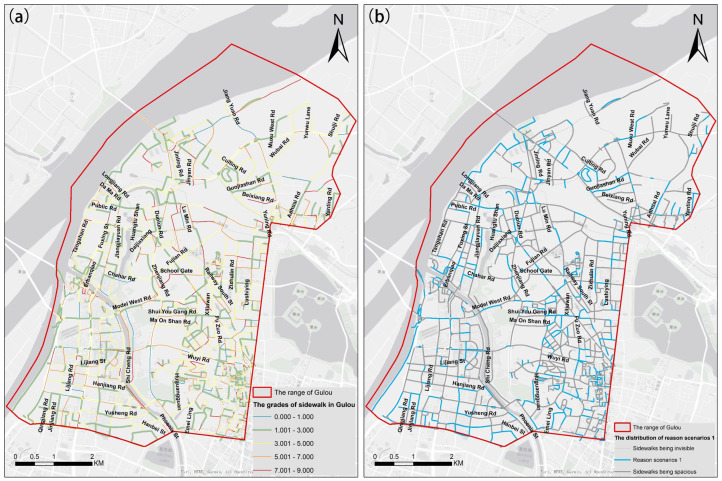
The distribution of sidewalks grades in Gulou (**a**) and the distribution of reason scenarios 1 (**b**).

**Figure 13:**
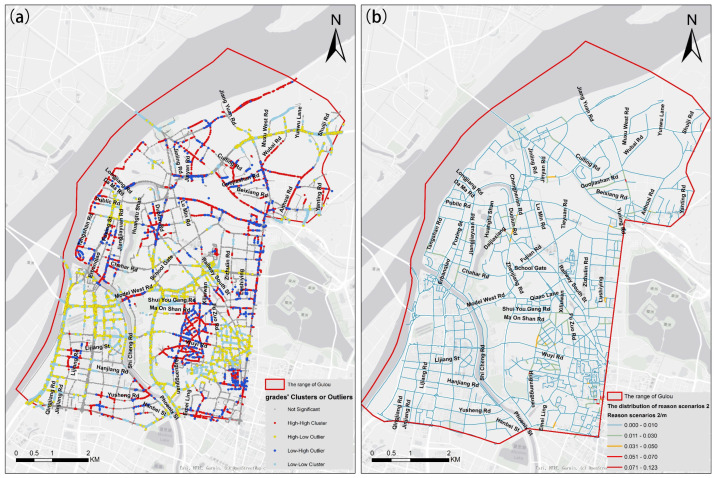
The LISA figure is based on spatial autocorrelation analysis on the grades of the sidewalk (**a**) and the occurrence frequency of reason scenario 2 in each road (**b**).

The sidewalk grade in every street view image is calculated using the above calculation method. Two types of RS and their distribution characteristic are extracted, as for HS1.RS1, we calculated the average of the sidewalk grades on each road, and the result is shown in Figure 12a. To better visualize the spatial distribution of sidewalk resources, we have used the blue sections in Figure 12b to represent the spatial distribution of HS1.RS1. As for HS1.RS2, we performed spatial auto-correlation analysis on the sidewalk grades in all images and obtained a LISA figure, as shown in Figure 13a. Among the four types of cluster/outliers, the Low-High Outlier is considered the HS1.RS2 and its spatial statistical results on each road are shown in Figure 13b.

It is found that the phenomenon of narrow sidewalks in the Gulou District is common in Figure 13a, accounting for 41.00% of the total roads. In Figure 13b, the local connectivity of the non-motorized transportation network in the southeastern Gulou District is directly limited by Fuzhou Road and Wuyi Road. Moreover, in the northwest and northeast of Gulou District, some roads are causing poor local connectivity, such as Duolun Road, Daijiaxiang Road, Guojiashan Road, and Yunning Road. It is vital to improve the linkage of non-motorized transportation to ensure pedestrians’ safety in the above areas.

### 3.2. Experimentation with Target Detection Methods

The seed points spreading algorithm can detect all target objects in theory, but the following problems may arise in practice. First, the semantic segmentation in the previous stage may miss or wrongly classify the categories of some pixels, which brings errors in this target detection stage. Second, the objects of a target may be split by other objects such as streetlights, bins, etc. Third, traversing the neighborhood points of each seed point would take much more time than standard target detection algorithms.

To evaluate the effectiveness of this method, comparison experiments have been carried out on this algorithm with Yolov4 and Yolov5 models. Since these two YOLO models are controversial in terms of performance comparison, the Yolov4 and Yolov5 are both used as the comparative models in this study. These two models have been trained and tested on two datasets, VOC2007and VOC2012 (https://pjreddie.com/projects/pascal-voc-dataset-mirror/, accessed on 7 June 2007 and 18 May 2012) and COCO (https://cocodataset.org/#home, accessed on 8 September 2017). The Yolov4 obtains the mean average precision (mAP) 0.5 score of 89% and this index for Yolov5 is 53.00%. In the comparison experiments, 50 street view images and their annotated images randomly selected from the Cityscape dataset are used to evaluate the models’ performance. The recall index, which is calculated as the proportion of detected target objects to all target objects in the image, and precision, calculated as the proportion of real ones to detected target objects, are used as the evaluation indicators. When calculating the evaluation indicators, we collectively refer to pedestrians and cyclists as non-motorized moving participants. The actual evaluation results of the two algorithms are compared in Table 6.

The seed points spreading algorithm, Yolov4 and Yolov5 all achieve promising precision, which means only a minority of the target objects identified by these two algorithms are unreal objects. However, these algorithms differ greatly in the recall index, and the recall indexes of Yolov4 and Yolov5 are much lower than that of the seed points spreading algorithm, which indicates that the Yolo models may miss plenty of target objects when processing the panoramic images. However, the evaluation result does not indicate that the seed points spreading algorithm is superior to Yolo models in any research background, since the algorithm is extremely time-consuming to run, and the cost of time spent increases significantly with the types of the target object. Furthermore, the performance of this algorithm greatly depends on the performance of semantic segmentation model. Due to the errors generated during semantic segmentation process, several ground bricks, warning signals and other ground objects are incorrectly detected as pedestrians, which have a negative impact on the subsequent scenario screening. As a result, the seed points spreading algorithm and PSPNet model are used for target detection and primary screening for three categories of scenarios. Furthermore, we adopt the Yolov4 model which has better comprehensive performance to re-identify whether the suspected hazardous scenarios contain pedestrians or cyclists, and to eliminate those fake ones.

### 3.3. Image Classification Experiments Based on SENet

SENet model is trained on the pre-trained model and implemented image classification on the suspected scenarios to eliminate the false and retain the true. The epoch of each training process is set to 300, and the batch size is set to 5. The SGD is used as the optimizer, while the learning rate is set to 0.045 at the beginning and decreases during training, and momentum rate is set to 0.9.

A total of five groups of comparison experiments are conducted, each of which assesses the performance of SENet’s binary classification on HS1, HS2, and HS1.RS3. In experiment 1, the data inputted into the SENet model is an RGB-channels image with three channels. In experiment 2, the input data is a single channel semantic segmentation image. In experiment 3, the semantic segmentation image and RGB-channels image are combined as input data with four channels. In experiment 4, the four-channel input data contains the grayscale image reflecting zebra lines as well as the RGB-channels image. In experiment 5, the data input into the SENet model is a combination of the various layers mentioned above, with a total number of channels of 5. The accuracy is employed as an indicator to evaluate SENet’s performance in binary classification, which indicates the proportion of correctly classified samples to the total number of samples. In the testing process, the absolute accuracy of SENet is shown in Table 7.

The five groups of experiments prove that SENet can adapt to multi-channel input data. For HS1 and HS1.RS3, the accuracy of experiment 5 achieves the highest. For HS2, experiment 1 has the highest accuracy, which means using RGB channel images as input data is more favorable for SENet to perform this classification task. In addition, it is believed that distinguishing HS2 does not need to consider environmental elements such as sidewalks, motor vehicle lanes, and zebra lines. However, the five-channel input data in experiment 5 make the model’s classification performance closer to the results in experiment 1. Although we cannot decipher the inherent judgment mechanism of the deep learning model, it is evident that SENet’s ability to filter useless information has been enhanced as the input information’s dimension increases. In other words, the semantic segmentation images and the grayscale image reflecting zebra lines have counteracted each other’s adverse effects.

**Figure 14:**
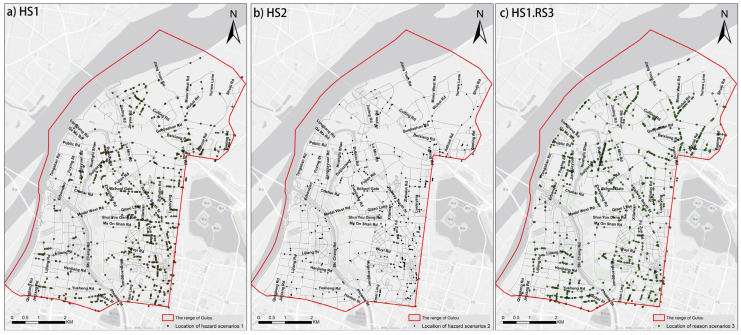
The distribution of various types of scenarios.

**Figure 15:**
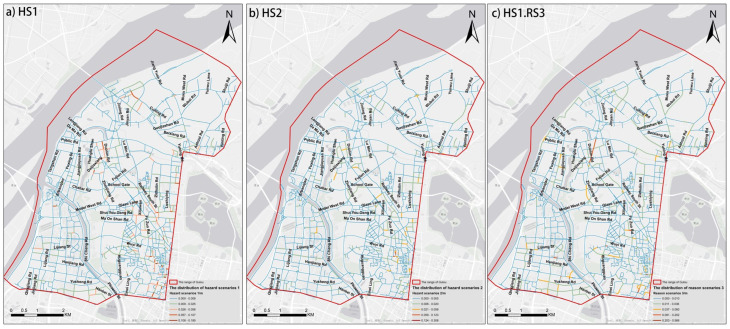
The occurrence frequencies of various types of scenarios in each road.

After combining each RGB-channel image of the cropped results with its grayscale image and the semantic segmentation image, we utilize the thoroughly trained SENet models to obtain the positive samples from each category of scenarios separately. Next, 1222 scenes for HS1, 479 scenes for HS2, and 1454 scenes for HS1.RS3 are obtained in the range of the Gulou district in Figure 14. To compare the severity and regional characteristics of non-motorized transportation safety threats in each area, the 10-m buffers of roads are also applied to perform spatial statistics on the three types of scenarios, as shown Figure 15.

Based on Figure 14a and Figure 15a, two relatively prominent characteristics of HS1 are concluded from a spatial perspective. Firstly, the POIs corresponding to HS1 continually occurs along the roads. Secondly, the POIs corresponding to HS1 are not randomly distributed within the study area range but concentrated within most of the southeastern regions and the northeastern and northeast parts of Gulou. We believe that the groups of residents are forced to adapt to the unfriendly non-motorized traffic environment and thus collectively step into the motor vehicle lines. In Figure 14b and Figure 15b, it is found that the number of HS2 is much less than that of HS1, and these HS2 are mainly clustered on specific road sections, such as Zhenjiang Road, School Gate, Emei Ling Road. In Figure 14c and Figure 15c, the distribution area of HS1.RS3 is more extensive, indicating that intensive obstacles are common in the Gulou District. By comparing the distribution of HS1.RS3 and HS1, the existing impediments on the sidewalk, make pedestrians more dangerous, such as Yusheng Road, Hanbei Street, Fuzhou Road, and Guangzhou Road.

### 3.4. Countermeasures for HS

For solving HS1 and improving the non-motorized transportation environment, three strategies are proposed: urban planning guidance, facility improvements, and management enhancement. In terms of urban planning guidance, the government should give full play to the leading function of the master plan and require the systematization, connectivity and practicality of non-motorized transportation in the form of regulations. When formulating the relevant plans, the sidewalks at the location of the Low-High outlier points should be prioritized for rectification, which will help enhance the connectivity of the existing spacious sidewalks with minimum economic and land costs. As for the improvements of facilities, based on possible traffic conflicts among multiple types of transportation participants, it is recommended that consideration be given to reallocating road space rights between motorized and non-motorized traffic participants. In other words, compress the space of motor vehicle lanes to make space for cyclists and require the width of the non-motorized road to be not less than 2 m.

**Figure 16:**
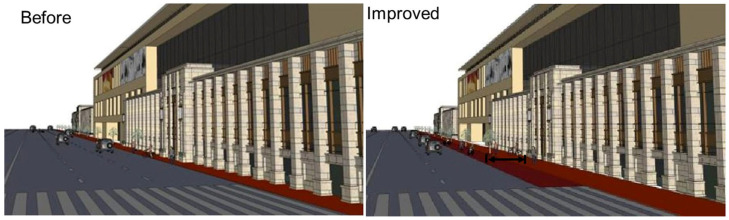
Comparison of the road environment.

**Figure 17:**
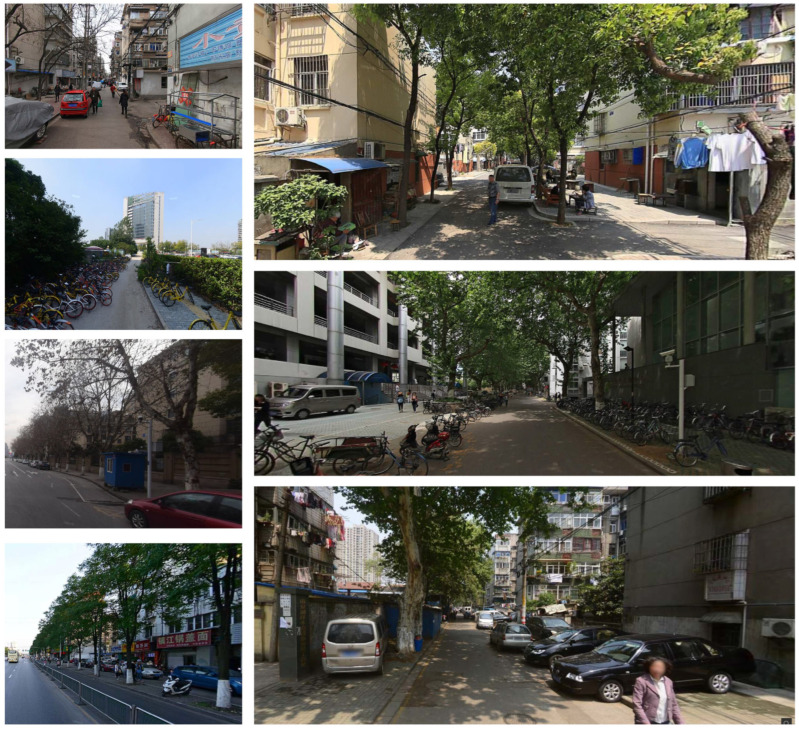
The representative scenes for HS1.RS3.

Moreover, it is suggested that the non-motorized lanes and sidewalks need to be significantly separated from motorized routes through height differences to ensure the safety of pedestrians, as shown Figure 16. Regarding enhancing management, we find that the illegal occupation of sidewalk space by stores and vendors also dramatically affects the sidewalk space, as shown Figure 17. For solving this problem, the generally adopted approach is to arrange administrative staff patrol along the streets and alleys to clear the obstacles.

**Figure 18:**
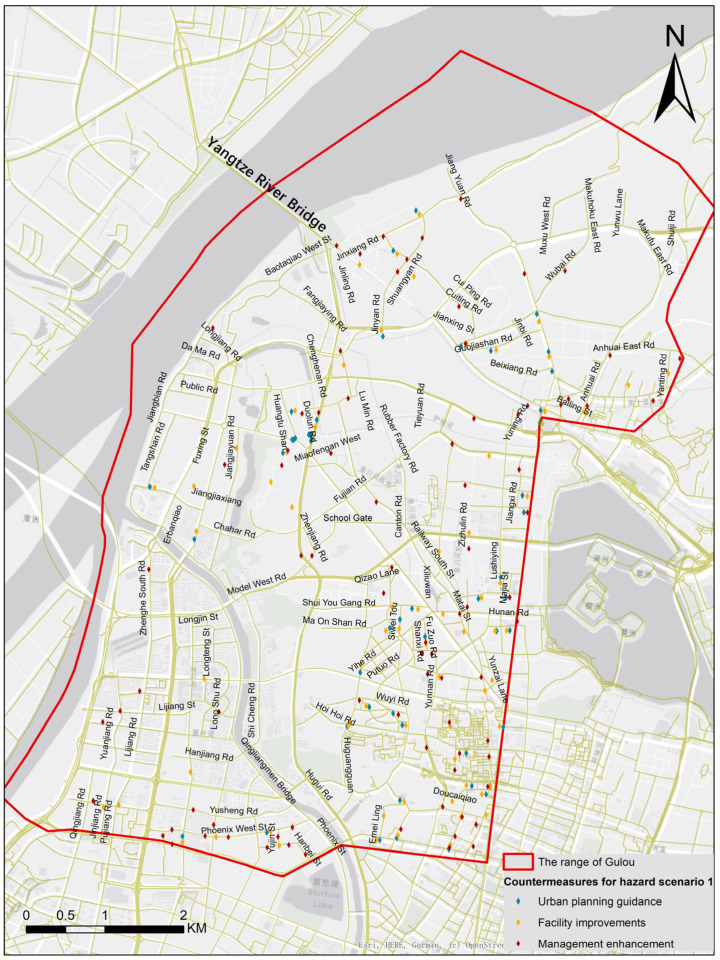
The countermeasures for HS1 in specific road sections.

We believe that planning guidance strategies and facility improvement strategies should be implemented in areas where both HS1 and HS1.RS2 are dense. In other words, priority should be given to the sections where pedestrian travel safety is a more complex situation or to areas that contribute to the connectivity and resilience of the sidewalk network in the Gulou District. For areas where both HS1 and HS1.RS3 are relatively dense, and it is recommended that an enhanced management strategy be implemented. Urban management departments can adjust their daily patrol routes to clear the sidewalks of occupied vendors and obstacles such as bicycles on time. Based on the spatial distribution characteristics of HS1 and three types of RS, we suggest that the corresponding improvement strategies be implemented for the road sections in Figure 18.

**Figure 19:**
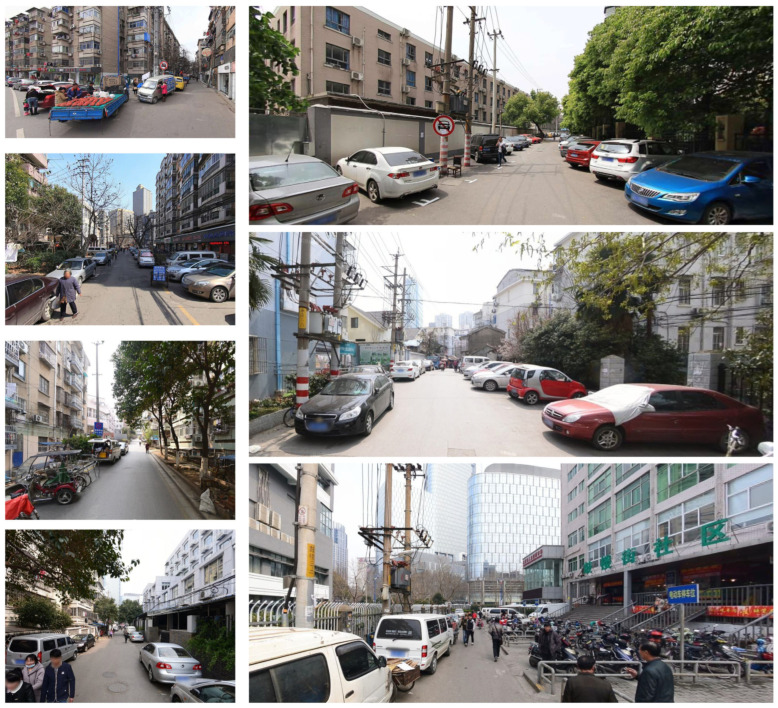
The representative scenes for HS2 are caused by motor vehicle and bicycle parking.

**Figure 20:**
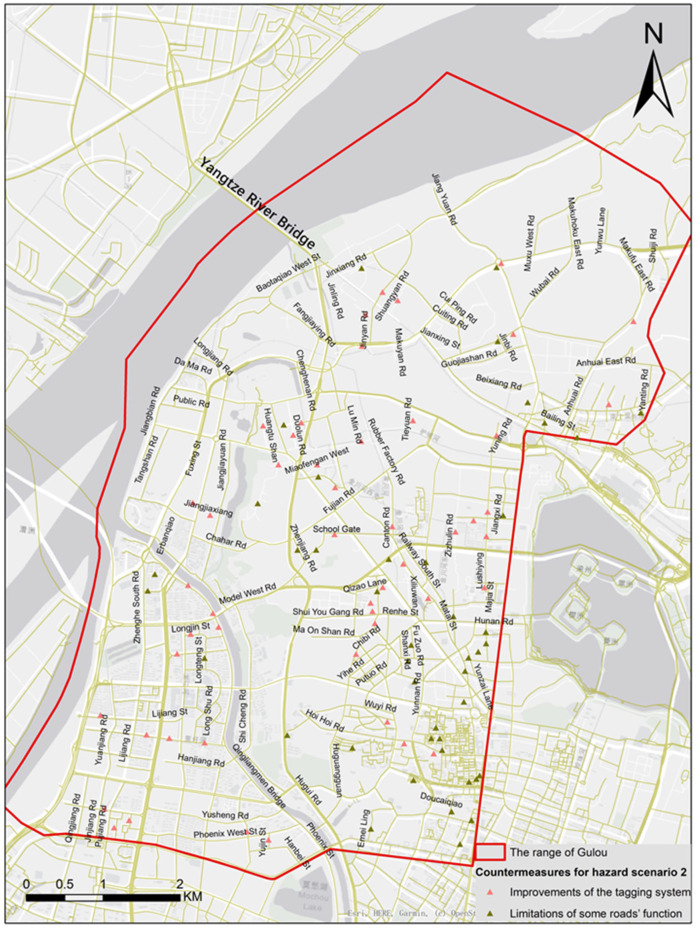
The countermeasures for HS2 in specific road sections.

This study also provides two solutions for solving HS2: improvements to the tagging system and the limitations of some roads’ functions. First, the government should put safety reminder tags in places where dangerous scenes frequently occur to remind pedestrians to walk close to the roadside. In addition, the automatic identification method of hazardous scenarios can be integrated with the urban road monitoring system to extract uncivilized behaviors from the road surveillance video. Furthermore, it is also found that many branch roads of Gulou District serve the function of walking, cycling, and motor vehicle driving and are used by nearby residents as open parking lots for motor vehicles and bicycles, as shown in Figure 19. Meanwhile, the visual blind spots when cars start, and stop may also pose new safety hazards for residents. Therefore, the government should designate these branch roads as No-Parking Zones for full time or specific periods, according to the actual situation of these roads, such as traffic flow, amounts of pedestrians and other information. Based on the spatial distribution characteristics of HS2, we suggest that the corresponding improvement strategies be implemented for the road sections in Figure 20.

## 4. Conclusions

In this study, a comprehensive processing method based on multiple deep learning models (Seed Points Spreading algorithm + PSPNet and SENet) is designed to automatically identify the most intuitive and objective hazard scenarios relevant to non-motorized transportation. The extracted information is used to assess and improve non-motorized transportation safety situations using the street view images in Gulou District, Nanjing.

Based on the width information of sidewalks and their spatial distribution characteristic, HS1.RS1 and HS1.RS2 are identified. It is also clearly found that the problem of discontinuous sidewalk facilities or narrow sidewalk widths is prevalent in Gulou District. After that, the seed points spreading algorithm is used to target detection for HS1, HS2 and HS1.RS3. Compared with Yolov4 Yolov5, the seed points spreading algorithm achieves promising precision. In addition, the recall of the algorithm is much higher than Yolov4 and Yolov5. In addition, the seed points spreading algorithm breaks the functional limitation of target detection and semantic segmentation to a certain extent, providing new ideas for future research on deep learning models. Next, the SENet is used to identify HS1, HS2 and HS1.RS3. and there are 1222 scenes for HS1, 479 scenes for HS2, and 1454 scenes for H1.RS3 in the range of Gulou District. We provide corresponding solution strategies for each area based on the spatial clustering characteristics of the above study scenarios.

For solving HS1 and improving the non-motorized transportation environment, three strategies are proposed: urban planning guidance, facility improvements, and management enhancement. To solve HS2, the government should put safety reminder tags in places where dangerous scenes frequently occur to remind pedestrians to walk close to the roadside. Furthermore, these branch roads should be designated as No-Parking Zones for full time or specific periods, according to the actual situation of these roads, such as traffic flow, amounts of pedestrians and other information.

This study still has many shortcomings. For example, the research results cannot be used as information support for real-time management. The street view images presented on the Baidu map platform always lag the actual occurrence of conflicts. However, this study has proposed a complete method to extract various scenarios from ordinary RGB-channels images. We will try to apply this method to real-time images collected from cars recorders and road surveillance facilities. These data sources make it possible to detect safety hazards in real-time traffic environments, which can help to alert motorists to take the initiative to slow down and prevent danger.

## Figures and Tables

**Table 1:** The description of various categories of study scenarios.

Category	Road Environment	Description of Scenario
HS1	There is a clear division of sidewalks and motor vehicle lane	A pedestrian walking in the motor vehicle lane
HS1.RS1		Sidewalks being excessively narrow
HS1.RS2		Sidewalks being spatially discontinuous
HS1.RS3		Bicycles and other obstacles occupy the sidewalks
HS2	There is no clear division of sidewalks and motor vehicle lanes	Motor vehicles, non-motor vehicles and pedestrians mixing in close distance

**Table 2:** The pixel values and corresponding categories in annotated images of the Cityscape data.

Value	0	1	2	3	4	5	6
Category	Motorway	Sidewalk	Building	Wall	Fence	Pole	Traffic light
Value	7	8	9	10	11	12	13
Category	Traffic sign	Vegetation	Terrain	Sky	Person	Rider	Car
Value	14	15	16	17		18	
Category	Trunk	Bus	Train	Motor cycle		Bicycle	

**Table 3:** The specific parameter setting of convolution layer and pooling layer in PSPnet.

Layer Name	Parameter Configuration	Description	Module Location
Conv1_x	3 × 3, 64 (Conv layer) 3 × 3, 64 (Conv layer) 3 × 3, 128 (Conv layer) 1 × 1, 256 (Conv layer)		ResNet convolution module [34]
Max_Pool1	3,3,2,2 (pooling layer) 1 × 1, 256 (Conv layer)		
Conv2_x	1 × 1, 64 (Conv layer) 3 × 3, 64 (Conv layer) 1 × 1, 256 (Conv layer)	Repeat this block 3 times	
Conv3_x	1 × 1, 128 (Conv layer) 3 × 3, 128 (Conv layer) 1 × 1, 512 (Conv layer)	Repeat this block 4 times	
Conv4_x	1 × 1, 256 (Conv layer) 3 × 3, 256 (Conv layer) 1 × 1, 1024 (Conv layer)	Repeat this block 23 times	
Conv5_x	1 × 1, 512 (Conv layer) 3 × 3, 512 (Conv layer) 1 × 1, 2048 (Conv layer)	Repeat this block 3 times	
Avg_Pool2	15,15,15,15 (pooling layer) 30,30,30,30 (pooling layer) 45,45,45,45 (pooling layer) 90,90,90,90 (pooling layer)		pyramid pooling module
Conv5_4	3 × 3, 512 (Conv layer)		outputting module
Conv6	1 × 1, 18 (Conv layer)	18 equals to the total number of classes	

**Table 4:** The screening criterion for each type of suspected scenarios.

Category	Rules for the Recognition
HS1	(a) the lowermost contour of the pedestrian intersects with the motor vehicle lane
HS2	(a) the outer rectangle of the cyclist intersects the outer rectangle of a motor vehicle.
	(b) the outer rectangle of the pedestrian intersects the outer rectangle of a motor vehicle.
	(c) the outer rectangle of the pedestrian intersects the outer rectangle of the cyclist.
HS1.RS3	(a) the outer contour of motor and non-motor vehicles intersects the sidewalk.

**Table 5:** The specific parameter setting of convolution layer and pooling layer in ResNet embedded in SENet [34].

Layer Name	Parameter Configuration	Description
Conv1_x	7 × 7, 64 (Conv layer)	
Max_Pool1	3,3,2,2 (pooling layer)	repeat this block 1 time;
Conv2_x	1 × 1, 64 (Conv layer) 3 × 3, 64 (Conv layer) 1 × 1, 256 (Conv layer)	repeat this block 3 times;
Conv3_x	1 × 1, 128 (Conv layer) 3 × 3, 128 (Conv layer) 1 × 1, 512 (Conv layer)	repeat this block 4 times;
Conv4_x	1 × 1, 256 (Conv layer) 3 × 3, 256 (Conv layer) 1 × 1, 1024 (Conv layer)	repeat this block 6 times;
Conv5_x	1 × 1, 512 (Conv layer) 3 × 3, 512 (Conv layer) 1 × 1, 2048 (Conv layer)	repeat this block 3 times;
Avg_Pool2	7,7,1,1 (Conv layer)	
Full Connect (FC)		Input the feature map output from Avg_Pool2 to this Full Connection layer.

**Table 6:** The precision and recall of each group of comparative experiments.

	Seed Points Spreading + PSPNet		Yolov4		Yolov5	
	NMP	BB	NMP	BB	NMP	BB
Precision	82.69%	90%	98.46%	95.00%	98.71%	100.00%
Recall	92.45%	100%	23.61%	20.88%	33.04%	9.80%
Time	4.53 s		1.73 s		1.51 s	

Note: (NMP) non-motorized moving participants; (BB)Bicycle/electric bicycle.

**Table 7:** Testing accuracy of each group of comparison experiments.

	Experiment1	Experiment2	Experiment3	Experiment4	Experiment5
HS1	67.14%	69.61%	72.08%	75.27%	76.09%
HS2	90.33%	84.01%	83.27%	84.76%	89.38%
HS1.RS3	73.20%	69.93%	79.74%	80.07%	83.08%

## Data Availability

The data in this study can be availed in the article.
